# Subpopulations of AIB1 isoform-expressing breast cancer cells enable invasion and metastasis

**DOI:** 10.18632/oncotarget.28452

**Published:** 2023-08-30

**Authors:** Amber J. Kiliti, Ghada M. Sharif, Anton Wellstein, Anna T. Riegel

**Keywords:** AIB1, AIB1Δ4, breast cancer, invasion, metastasis

Genetic and epigenetic events drive individual tumor cells to proliferate and expand into a heterogeneous mixture of cells that evade immune surveillance, acquire the ability to invade the vasculature and spread as metastatic seeds to distant sites. Organ metastasis contributes to more than 90% of all cancer related deaths [1]. The model of Darwinian evolution explains the stepwise selection of cancer cells capable of invasion and metastatic spread and an extensive body of work supports that cancer cell-autonomous features match the selected cancer cell “seed” with the appropriate “soil” of the target organ. However, this concept was challenged in a recent paper in Cancer Research [2]. Sharif et al. observed that a subclonal population of cells in a heterogeneous tumor can significantly alter the growth characteristics, invasiveness and metastasis of an entire tumor through cell-cell crosstalk. These functionally relevant cell subpopulations are difficult to detect through bulk analysis though their presence may influence disease outcome and efficacy of treatments. In their paper, Sharif et al. detailed how expression of a splice isoform of the transcriptional coregulator and oncogene Amplified In Breast Cancer 1 (AIB1) in a small subpopulation of cells can lead to increased tumor growth and invasion of surrounding tissues by ductal carcinoma *in situ* (DCIS) cells. Interestingly, this subpopulation did not become the dominant population during malignant progression but remained present at low levels in both primary and metastatic tumors. The authors focused on malignant progression of DCIS, a non-invasive stage of breast cancer that accounts for 20% to 30% of newly diagnosed breast cancer cases in the United States [3]. DCIS has the potential to acquire an invasive and metastatic phenotype but the drivers of this transition are largely unknown.

Sharif et al. CRISPR-engineered the immortalized normal human mammary epithelial MCF10A cells and their derivative early-stage breast cancer cells (MCF10DCIS) to only express a naturally occurring splice isoform of the AIB1 oncogene. In the AIB1Δ4 isoform, exon 4 is spliced from the full-length mRNA, resulting in the loss of the first 223 amino acids from the N-terminus compared to the full length AIB1 protein. AIB1Δ4 expression increases with malignant progression, loses interactions with transcription corepressors [4, 5] and is a potent transcriptional coactivator [6]. Mixing of the AIB1Δ4-expressing cells (DCISΔ4) with the non-invasive parental DCIS cells at a 1:4 ratio (majority parental cells) showed cell-cell crosstalk between the two populations that led to increased invasion of the parental cells, a phenotype they termed “enabling”.

The authors initially observed this enabling phenotype in 3D growth assays *in vitro* showing increased invasion into the surrounding mixture of collagen and matrigel by parental DCIS cells in the presence of DCISΔ4 cells. This phenotype persisted *in vivo*. Mice inoculated with a mixture of DCIS with a minor subpopulation of DCISΔ4 cells grew larger tumors and developed lung metastases earlier compared to mice inoculated with DCIS cells alone. Surprisingly, the DCISΔ4 cells were only able to form small heterogeneous and less differentiated DCIS lesions when injected alone.

Sharif et al. showed that DCIS and DCISΔ4 cells differed in their transcriptomes through RNA-sequencing experiments. Inflammatory response genes and extracellular matrix proteases were upregulated in the DCISΔ4 cell line compared to the parental DCIS cells. By creating a signature gene set with the top up- and down-regulated genes in the DCISΔ4 cells, the authors found that breast cancer patients that had altered expression of these genes had worse relapse-free and overall survival. Furthermore, they showed that co-culturing these cells and then separating them by flow cytometry prior to RNA-sequencing revealed differentially expressed genes compared to baseline expression. Notably, DCIS cells co-cultured with DCISΔ4 cells had a significant increase in NF-κB and WNT signaling compared to DCIS cells cultured alone, highlighting the impact of cell-cell crosstalk and its probable importance for the enabling phenotype.

As an explanation for the enabler phenotype, the authors uncovered differential genomic engagement of the AIB1Δ4 isoform compared to AIB1 through ChIP-sequencing. They found that AIB1Δ4 had a distinct cistrome compared to full length AIB1. AIB1Δ4 was uniquely enriched at PPARRE (peroxisome proliferator-activated receptor response elements) motifs and GRE (glucocorticoid response elements) was the top enriched motif in DCISΔ4 cells. Using this information, Sharif et al. were able to block the enabling effect of AIB1Δ4 by activating the PPARγ signaling pathway with the agonist efatutazone. Conversely, the enabling effect was enhanced when activating the glucocorticoid receptor with dexamethasone. Further experiments are needed to decipher the precise mechanism of enabling but the authors provide insights as to a central role for nuclear receptor signaling.

**Figure 1:**
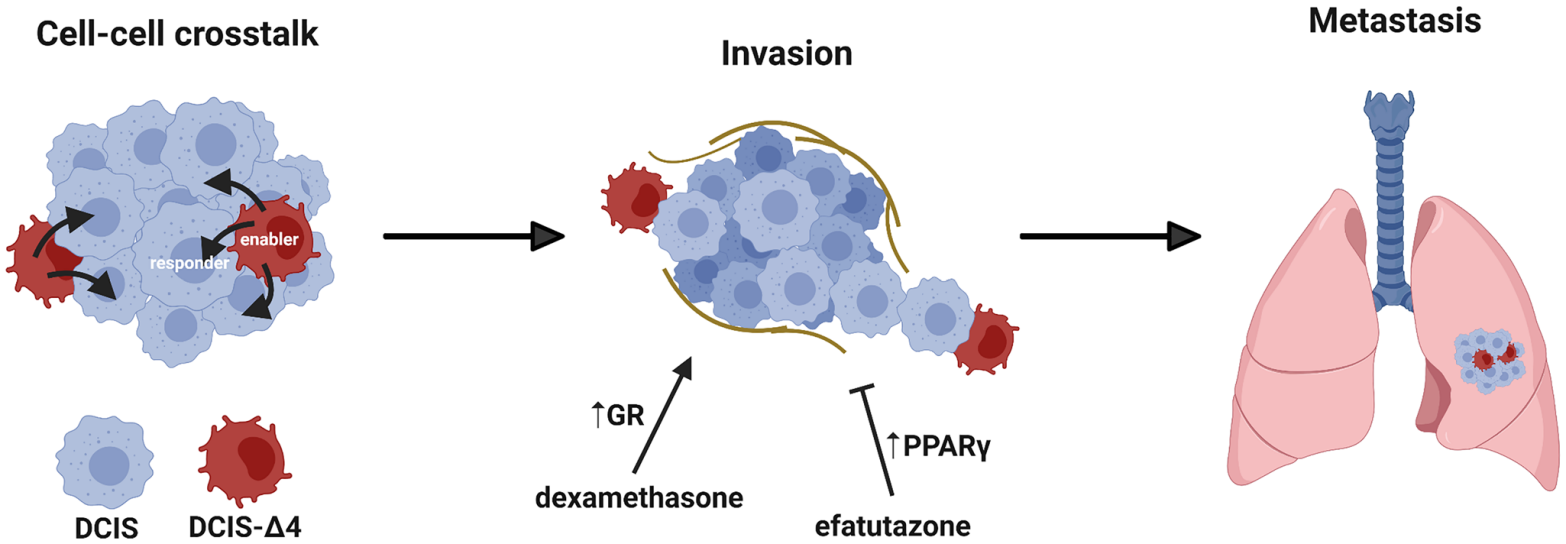
Graphical representation of AIB1Δ4 enabling DCIS invasion and metastasis. A subpopulation of AIB1Δ4-expressing cells enables DCIS invasion and metastasis through cell-cell crosstalk that is in part dependent on GR and PPARγ signaling.

AIB1Δ4 is found at increased levels in high-grade compared to low-grade DCIS. The data in this paper suggest that even a small subpopulation of AIB1Δ4-expressing cells can enable cell invasion through inducing field effects on signaling pathways of neighboring tumor cells (Figure 1), making AIB1Δ4 a driver of invasive progression of DCIS and a potential clinical biomarker of disease progression. Pharmacologically targeting pathways that are selectively activated through cellular crosstalk holds promise for inhibiting enabling. Additional studies aimed at mechanistically characterizing how AIB1Δ4 expression leads to enabling will hold significant clinical relevance for blocking progression of *in situ* to invasive cancer that may ultimately curtail the metastatic progression of disease.
